# Safety and efficacy of autologous cell vaccines in solid tumors: a systematic review and meta-analysis of randomized control trials

**DOI:** 10.1038/s41598-023-29630-9

**Published:** 2023-02-27

**Authors:** Donald J. Bastin, Joshua Montroy, Michael A. Kennedy, Andre B. Martel, Risa Shorr, Maryam Ghiasi, Dominique M. Boucher, Boaz Wong, Louise Gresham, Jean-Simon Diallo, Dean A. Fergusson, Manoj M. Lalu, Natasha Kekre, Rebecca C. Auer

**Affiliations:** 1 Cancer Therapeutics Program, The Ottawa Hospital Research Institute, General Campus, 501 Smyth Road, Ottawa, ON K1H 8L6 Canada; 2 Schulich School of Medicine, Western University, London, ON Canada; 3 Clinical Epidemiology Program, Blueprint Translational Research Group, Ottawa Hospital Research Institute, Ottawa, ON Canada; 4 Department of Surgery, University of Ottawa, Ottawa, ON Canada; 5 Faculty of Medicine, University of Ottawa, Ottawa, ON Canada; 6 Department of Biochemistry, Microbiology, and Immunology, University of Ottawa, Ottawa, ON Canada; 7 Learning Services, The Ottawa Hospital, Ottawa, ON Canada; 8 School of Epidemiology and Public Health, University of Ottawa, Ottawa, ON Canada; 9 Department of Anesthesiology and Pain Medicine, The Ottawa Hospital, University of Ottawa, Ottawa, ON Canada; 10 Regenerative Medicine Program, The Ottawa Health Research Institute, Ottawa, ON Canada

**Keywords:** Cancer, Surgical oncology, Cancer immunotherapy

## Abstract

We conducted a systematic review and meta-analysis of randomized control trials to formally assess the safety and efficacy of autologous whole cell vaccines as immunotherapies for solid tumors. Our primary safety outcome was number, and grade of adverse events. Our primary efficacy outcome was clinical responses. Secondary outcomes included survival metrics and correlative immune assays. We searched MEDLINE, Embase, and the Cochrane Central Register of Controlled Trials for studies published between 1946 and August 2020 using any autologous whole cell product in the treatment of any solid tumor. The Cochrane Randomized Controlled Trial risk of bias tool was used to assess risk of bias. Eighteen manuscripts were identified with a total of 714 patients enrolled in control and 808 in vaccine arms. In 698 patients receiving at least one dose of vaccine, treatment was well tolerated with a total of 5 grade III or higher adverse events. Clinical response was reported in a minority (n = 2, 14%) of studies. Autologous cell vaccines were associated with improved overall (HR 1.28, 95% CI 1.01–1.63) and disease-free survival (HR 1.33, 95% CI 1.05–1.67) over thirteen and ten trials respectively. Where reported, immune assays correlated well with clinical outcomes. Our results suggest that autologous whole cell vaccination is safe and efficacious in increasing survival in patients undergoing treatment for solid tumors.

*Registration*: PROSPERO CRD42019140187.

## Introduction

Surgical excision represents a cornerstone in the treatment of solid tumors. Despite significant advances in adjuvant therapies, disseminated disease remains a chief cause of mortality following operative management^1^. Immunotherapies have shown promising results in the treatment of certain solid malignancies and early studies suggest that they may provide benefit in the adjuvant setting following surgical treatment of solid tumors^2–4^.

Autologous whole cell vaccination represents one approach to immunotherapy in which a patient’s own tumor cells serve as a source of antigen. Following ex vivo isolation and manipulation, these cells are rendered replication defective and re-administered to the patient along with an immunogen, to produce an anti-tumor response in vivo. This approach has several theoretical advantages over other forms of immunotherapy. These include the breadth of antigenic coverage included in the vaccine, and the diversity of immune cell populations that can be recruited by such a product. Furthermore, unlike other approaches to cancer vaccination, the autologous product is highly specific to a patient’s tumor but does not require sequencing level knowledge of the cancer’s antigenic landscape^5,6^.

Numerous early-stage clinical trials have employed autologous whole cell vaccines as adjuvants to the surgical treatment of cancer. Currently, two autologous whole cell products are being investigated in this context in phase III clinical trials^7,8^.

While the results of these studies are eagerly anticipated, several other RCTs have been previously published showing positive results. Despite this, there is little formal consensus on the overall safety and efficacy of these products or in which disease they are most likely to be of benefit. In order to guide further development in this field, we have conducted a systematic review of randomized control trials investigating the use of autologous whole cell vaccines in the treatment of solid tumors. Our primary safety outcome was number and grade of adverse events while our primary efficacy outcome was clinical response. Secondary outcomes included survival metrics and correlative immune assays while tertiary outcomes focussed on health utility and economic factors. The results of this study serve to guide further investigations into the use of autologous whole cell vaccines in treating solid tumors and facilitate the design of future clinical trials.

## Methods

The results in this manuscript are presented in accordance with Preferred Reporting Items for Systematic Reviews and Meta-Analyses (PRISMA) guideline (Appendix 1)^9^. The protocol has been documented on PROSPERO (CRD42019140187) and previously published^6^. Our protocol was designed for use both in the current systematic review, as well as in a systematic review investigating the use of autologous cell vaccination in the treatment of hematologic maligancies^6^.

### Search strategy

The search strategy employed in this manuscript was developed in collaboration with an information specialist and clinical expert in the field and is available in Appendix 2. The search was applied to MEDLINE (OVID interface, including In-Process and Epub Ahead of Print), Embase (OVID interface), and the Cochrane Central Register of Controlled Trials (Wiley Interface) for articles published between 1946 and August 6th, 2020. Forward and reverse citation searching were also performed on any studies which were deemed to meet eligibility criteria^10^. No language restrictions were imposed on the search. Clinicaltrias.gov was not included in the search as a decision was made to focus on results of published RCTs.

### Eligibility criteria

Eligible studies were clinical trials which employed an autologous whole cell vaccine product in a solid malignancy. Any study employing an autologous whole cell product as part of the intervention was considered, however studies using only allogeneic or lysate vaccines were excluded. No restrictions were placed in terms of patient demographics, publication date, disease site, or prior/concurrent treatments. Only randomized controlled trials were considered for this review and single arm studies, non-randomized trials, and randomized trials without a control group were excluded. Similarly, only complete manuscripts detailing clinical trials were included. Case reports, conference abstracts, letters, reviews, and editorials were not considered.

### Study selection and data collection processes

Titles and abstracts of all studies captured by our search strategy were screened by two out of four possible reviewers independently (DJB, MAK, STK, ABM) for eligibility using Distiller Systematic Review Software (DistillerSR, Evidence Partners, Ottawa, Canada). Any conflicts were flagged by the software and reviewers met to resolve the conflict. In the event of two reviewers not reaching a consensus, a third reviewer was included to facilitate resolution. Studies that were deemed eligible at this point then proceeded to full text screening which was similarly carried out in duplicate using DistillerSR. Studies that were deemed eligible at both the levels of title/abstract and whole text screening were included in the review and proceeded to data extraction. Data extraction was performed in duplicate (DJB, MAK) using standardized forms in DistillerSR. Extracted data included study details (country and year of publication, recruitment period, follow up period, patients enrolled in treatment and control groups), patient characteristics (malignancy and stage), intervention details (fresh vs. frozen and fixed vs. irradiated vaccine, dosage and administration details, adjuvants used, and prior/concurrent interventions), adverse events reported, clinical response data, overall and disease-free survival, and results of immune assays. Prior to data extraction, forms were pilot tested by two independent reviewers (DJB, JM). As with study selection, conflicts were resolved first through discussion between reviewers and then by a third reviewer if a consensus was not reached.

### Risk of bias

Risk of bias was assessed by two independent reviewers (DJB, MAK) using the Cochrane Randomized Controlled Trial risk of bias tool^11^. Discrepancies were flagged by the Distiller software and reviewers met for conflict resolution. If a consensus was not obtained by two reviewers, a third party (JM) was used as a tie breaker.

### Outcomes

The primary outcomes assessed by this systematic review were safety and efficacy of the autologous cell products based on reported adverse events (AEs) and clinical response respectively. Clinical responses included complete response (CR) and overall response (OR). In our published protocol, clinical response was primarily intended to be applied to our companion study of autologous cell vaccination hematologic malignancies where CR and OR could be reported in the context of minimal residual disease^6,12^. We nevertheless recorded and reported on this outcome where it was reported in studies investigating solid tumors.

Secondary outcomes included survival metrics (overall and disease-free survival) and immune assays. Information on immune assays included whether specific assays including ELISpot, delayed type hypersensitivity (DTH), and intracellular cytokine staining (ICS) were performed and whether positive outcomes as defined by the studies were observed and corelated with clinical outcomes.

Tertiary outcomes were metrics of reported health-related quality of life, health utility, and biological assays that correlate with efficacy.

### Data analysis

Studies were pooled using Comprehensive Meta-Analyst (version 3; Biostat Inc., USA). For dichotomous outcomes (e.g. CR and OR), risk ratios were calculated using a random-effects analysis based on the Der-Simonian Laird model and presented with 95% confidence intervals. For time-to-event outcomes (i.e. disease-free and overall survival), hazard ratios were calculated using a random-effects generic inverse-variance model, and presented with 95% confidence intervals. Statistical heterogeneity was assessed using the I^2^ statistic. Thresholds for determining heterogeneity were as recommended by the Cochrane Handbook (with 30–60% representing moderate, 50–90% representing substantial, and 75–100% representing considerable heterogeneity)^13^. Publication bias was evaluated using funnel plots and Egger’s regression, where sufficient data were available (Cochrane recommends at least 10 studies)^13^. A priori subgroup analyses included cancer type, or by type of adjuvant administered.

### Deviations from published protocol

In our published protocol we initially considered all clinical trials using autologous whole cell vaccines in the treatment of solid tumors without specifying that the study be an RCT. Our team decided to focus only on randomized control trials as they would provide the most robust evidence. Due to the range in dates over which studies were published and difficulties in obtaining updated contact information we did not contact authors for missing information.

### Ethics approval and consent to participate

Ethics approval is not required for this systematic review as the review uses solely published literature.

## Results

### Study selection and characteristics

**Figure 1:**
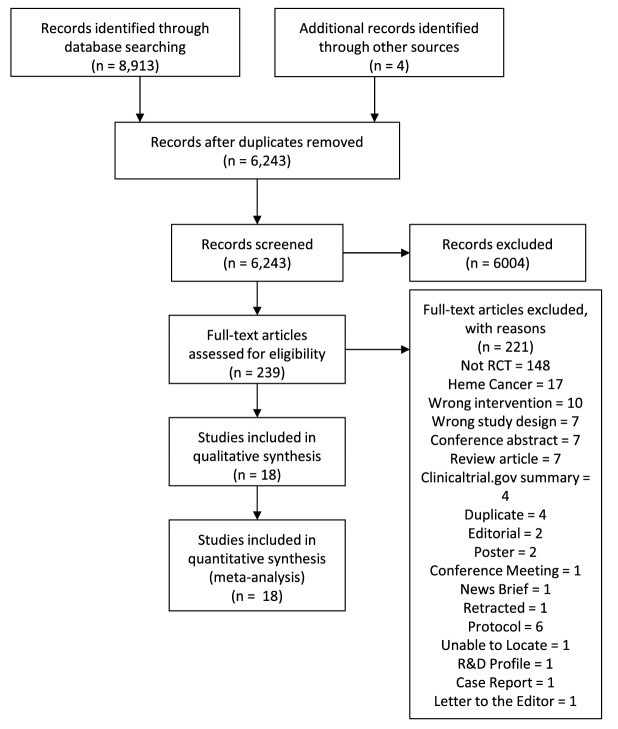
Study selection flow chart.

Our search strategy yielded 8913 citations representing 6243 unique manuscripts. After screening, a total of 14 randomized clinical trials met eligibility and an additional four reports were identified through forward and reverse citation searching and were deemed to meet eligibility criteria. Four of these studies were updates on trials reported by an earlier study captured in our systematic review. Thus, a total of 14 unique randomized clinical trials were included in our systematic review and meta-analysis (Fig. 1). In total there were 714 patients in the control groups, 808 patients enrolled to receive vaccination, and 698 being vaccinated with at least one dose. The characteristics of the trials are described in Table 1. In brief, studies spanned from 1977 to 2020 with half of the reports being published prior to 2000 and the other half in 2000 or after. The most common countries out of which trials were based were the United States (n = 4) and China (n = 3), with two trials occurring in the United Kingdom and one study in Italy, Germany, the Netherlands, Australia, and Israel respectively. The most frequent disease site was the colon/rectum (n = 5), followed by hepatocellular carcinoma (HCC) (n = 3), and renal cell carcinoma (RCC) (n = 2). One trial was performed in each of ovarian cancer, glioblastoma, lung cancer, and melanoma. Reported follow up times ranged from 12 months to a mean +/− SD of 116.1 +/− 23.8 months. In most studies, the patients were given the vaccine as an adjuvant following resection of the primary tumor. However, in the studies by Bota et al., and Embleton et al./McIllmurray et al., patients enrolled in the trial were undergoing surgery for a recurrence after previous therapy^14–16^. Similarly, Schulze et al. used an autologous cell product as an adjuvant following surgical resection of liver metastasis from a previously treated synchronous or metachronous colorectal tumor^17^. In the study by Hoover et al. some patients had had prior therapies while others had not^18^.

**Table 1:** Study characteristics.

Studies	Country	Multi-center?	Centralized manufacture protocol?	Malignancy	Stage	Patients (n)			Recruitment Period	Follow up period	Previous interventions	Vaccine name	Trial sponsor
						Control	Vaccine Group						
							enrolled	vaccinated					
Oh (2020) Oh (2016)	USA	Yes	Yes	Ovarian	III, IV+	11	20 (31)^°^	20 (31)^°^	2011–2015	Median 43.4mo (10.1–77.6mo)	No	Vigil®	Gradalis, Inc
Bota (2018)	USA	No	NA	Glioblastoma	IV+	4^†^	5^†^	5^†^	NR	NR	Surgery, chemotherapy, radiotherapy	ERC1671 (Gliova™)	Eiptopoietic Research Corporation
Schulze (2009)	Germany	Yes	NR	Colon	I–IV+	25	25	25	1991–1998	Mean (+/− SD): 116.1 ± 23.8mo vaccine, 112.4 ± 18.5 control	Surgery, chemotherapy, radiotherapy for synchronous / metachronous colon or rectal cancer	N/A	Unclear
Peng (2006)	China	No	NA	HCC	I, II	35	40	32	1999–2003	Median +/− SD: 33.6 ± 8.7	NR	N/A	Science Developing Plan Fund of Guandgong Province
Uyl-de Groot (2005), Vermorken (1999)	Netherlands	Yes	Yes	Colon	II, III	126	128	128	1987–1996	Median 5.8 years	No	OncoVAX	Intracel Corporation
Peng (2005)	China	No	NA	HCC	I–III	26	30	24	2000–2003	Median > 42 months	NR	N/A	Natural Science Foundation of Guangdong Province
Kuang (2004)	China	No	NA	HCC	I–III	21	19	18	2001–2003	Median 15 months (8–28)	NR	N/A	Ministry of Education, Culture, Sports, Science and Technology of Japan
Harris (2000)	United States	Yes	No	Colon	II, III	153	205	205*	1986–1993	Median 7.6 years	No	N/A	Public Health Service Grants (National Cancer Institute, National Institute of Health, Department of Health and Human Services) Intracel
Galligioni (1996)	Italy	NR	NR	RCC	I–III	54	60	60	1987–991	61 months	NR	N/A	Unclear
Hoover (1993)	United States	Yes	No	Colon / Rectum	I–III	39	50	41	1981–1990	Median 93 months (colon), 57 months (rectal)	None within 5 years of enrolment	N/A	National Institutes of Health Organon Teknika/Biotechnology Research Institute
Gray (1989, 1988)	Australia	Yes	Yes	Colon / Rectum	II, III	145	148	129	1978–1981	5 years	NR	N/A	Unclear
Adler (1987)	Israel	NR	NR	RCC	I–IV+	19	24	24	1980–1985	Median 30 months	NR	N/A	Frieda and Shmuel Sharfhartz Memorial Cancer Research Fund
Souter (1981)	United Kingdom	NR	NR	Lung	I, II	49	46	34	NR	NR	No	N/A	Cancer Research Campaign Grant
Embleton (1978), McIllmurray (1977)	United Kingdom	No	NA	Melanoma	II	7	8	8	NR	12 months	No	N/A	Cancer Research Campaign Grant

*NA* not applicable, *NR* not reported, *HCC* Hepatocellular Carcinoma, *RCC* Renal Cell Carcinoma.

^°^In the study by Oh (2016), 20 patients were randomized to the treatment group and 11 to the control; subsequently 10 additional patients were de-randomized and placed into treatment group. Our survival analysis focusses only on those who were randomized.

^†^Numbers from interim analysis.

*Although 205 patients were vaccinated, only 150 patients were deemed “analyzable” based on adequacy of vaccines and sufficient follow up information.

### Intervention details

In all trials the primary intervention used was surgery (Table 2). In twelve out of fourteen trials, the vaccine was given exclusively as an adjuvant following surgery with curative intent. In the study by Adler et al. an autologous vaccine product was given as an adjuvant to patients with stage III or lower renal cancer while the vaccine represented a palliative measure in surgically treated patients with stage IV disease^19^. In the trial described by Bota et al., patients had glioblastoma that was treated with surgery in which an R2 resection (gross residual tumor) was performed^14^. Twelve of fourteen studies specified whether a fresh or frozen product was given, and in all 12, the vaccine had been frozen prior to administration. Eleven trials specifically reported irradiating cells prior to administration whereas the remaining three trials employed a fixed cell product. Only three studies reported experiencing issues with vaccine quality, however 10 studies did not report on this metric. In all but one trial (which also employed endolymphatic installation), vaccines were administered exclusively through a subcutaneous route. Reported doses ranged from 0.1 to 100 × 10^6^ cells per dose, with numbers of doses administered ranging from 1 to 24. No study reported on a correlation with number of doses and outcomes. The bulk of the studies employed BCG (six trials) or GM-CSF (five trials) with alternative or additional adjuvants being miRNA engineered into the vaccine, Newcastle Disease Virus (NDV), Interleukin-2 (IL-2), *Vibrio cholerae* neuraminidase (VCN), and *C. parvum*. In three trials the vaccine was given alongside another therapy including chemotherapy, radiation, or hormone therapy.

**Table 2:** Intervention Details.

Studies	vaccine manufacturing considerations				Vaccine administration						Vaccine adjuvant		Other interventions	
	Fresh vs frozen	Irradiated cells?	Fixed cells?	Quality control issues?	Lowest dose (E6 cells)	Highest dose (E6 cells)	# Doses			Administration route	Identity	Incorporation strategy	Primary intervention	Concurrent therapies
							Intended	Provided						
Oh (2020) Oh (2016)	Frozen	Yes	No	NR	4	10	4–12	mean 7.8		Subcutaneous	GM-CSF; TGFβ targeting miRNA	Transfection	Surgery + Chemotherapy	None
Bota (2018)	Frozen	Yes	No	NR	0.1	1	2 autologous cells, 3 allogeneic cells	2 autologous cells, 3 allogeneic cells*		Subcutaneous	GM-CSF, CPA, allogenic lysate	Mixed with vaccine	Surgery	Chemotherapy
Schulze (2009)	Frozen	Yes	No	NR	10		6		6 (18 patients), 7 (1 patient), 11 (1 patient), 12 (1 patient), 3 (2 patients), 4 (1 patient), 5 (3 patients)	Subcutaneous	NDV	Infection (non-replicating)	Surgery	None
Peng (2006)	Frozen	NR	Yes	NR	NR; dose consists of 40uL of cells		3		3	Subcutaneous	GM-CSF, Il-2 microspheres, tuberculin	Mixed with vaccine	Surgery	None
Uyl-de Groot (2005), Vermorken (1999)	Frozen	Yes	No	NR	10		4		4 (101 patients), 3 (1 patient), 1 (1 patient), unknown (25 patients)	Subcutaneous	BCG	Mixed with vaccine	Surgery	None
Peng (2005)	Frozen	NR	Yes	NR	NR; consists of 10uL of cells		3		3 (24 patients), unspecified (6 patients)	Subcutaneous	GM-CSF, Il-2, tuberculin	Mixed with vaccine	Surgery	None
Kuang (2004)	NR	NR	Yes	NR	NR; dose consists of 40uL of cells		3		Unclear	Subcutaneous	Il-2, GM-CSF	Mixed with vaccine	Surgery	None
Harris (2000)	Frozen	Yes	No	Yes	10		3		Unclear	Subcutaneous	BCG	Mixed with vaccine	Surgery	None
Galligioni (1996)	Frozen	Yes	No	NR	10		3		Unclear	Subcutaneous	BCG	Mixed with Vaccine	Surgery	None
Hoover (1993)	Frozen	Yes	No	Yes	10		3		4 (1 patient), 3 (40 patients), 1 (1 patient)	Subcutaneous	BCG	Mixed with Vaccine	Surgery	Radiation
Gray (1989, 1988)	Frozen	Yes	No	No	0.5		18		NR	Subcutaneous	BCG; VCN treatment of cancer cells	Scarification	Surgery	None
Adler (1987)	Frozen	Yes	No	NR	3	100	5–6		≤ 24 / Unclear	Subcutaneous & Endolymphatic Installation	BCG	Mixed with Vaccine	Surgery	Hormone Therapy
Souter (1981)	Frozen	Yes	No	Yes	20		1		1	Subcutaneous	*C. parvum*	Mixed	Surgery	None
Embleton (1978), McIllmurray (1977)	NR	Yes	No	NR	50		1		1	Subcutaneous	BCG	Mixed with Vaccine	Surgery	None

*GM-CSF* granulocyte macrophage colony stimulating factor, *TGFβ* tumor growth factor beta, *miRNA* microRNA, *NR* none reported, *CPA *cyclophosphamide, *NDV* Newcastle disease virus, *Il-2* interleukin 2, *VCN* vibrio-cholera neuraminidase.

*in the study by Bota et al.(2018) it was unclear how many cycles of the 2 autologous cell and 3 allogeneic cell vaccines were provided.

### Primary outcomes

#### Safety

Adverse event reporting for control arm patients was inconsistently reported and therefore no pooled analysis could be performed. The data for adverse events is therefore reported descriptively and in tabular format (Table 3). Overall, the vaccine products were well tolerated with a total of five adverse events grade 3 or greater. In the trial by Bota et al., four grade 3 adverse events were reported in patients in the treatment arm while eight were reported in the control arm^14^. These events were mostly neurological in nature including headaches and gait disturbances. In the study by Gray et al., one patient in the vaccine arm developed end stage renal failure^20,21^. Notably, there were no deaths associated with administration of the autologous cell products in any study. The most frequently reported single adverse event was local complications such as erythema or pruritis. Fever was also a commonly reported AE, while other AEs that were not local complications or fever included headaches, dizziness, nausea or lymphadenopathy. Some studies reported adverse events as number of events while others reported the total number of patients experiencing the event on any injection and others provided both metrics.

**Table 3:** Adverse events.

Study	Vaccinated patients (n)	Doses provided per patient	Serious (Grade 3+) adverse events (n of events)	All adverse events (n of events)			Number of patients experiencing any AE
				Total (n)	Local complication (n)	Fever (n)	
Oh (2020) Oh (2016)	31°	mean 7.8 (max 12)	None	93	93	0	NR
Bota (2018)^†^	5^†^	2 autologous cells, 3 allogeneic cells*	4	162	67	NR	NR
Schulze (2009)	25	6 (18 patients), 7 (1 patient), 11 (1 patient), 12 (1 patient), 3 (2 patients), 4 (1 patient), 5 (3 patients)	None	5	4	0	4
Peng (2006)	32	3	None	62	7	30	20
Uyl-de Groot (2005), Vermorken (1999)	128	4 (101 patients), 3 (1 patient), 1 (1 patient), unknown (25 patients)	None	NR	NR	NR	128
Peng (2005)	24	3 (24 patients), unspecified (6 patients)	None	72	72	0	24
Kuang (2004)	18	Unclear	None	NR	NR	0	18
Harris (2000)^±^	205	Unclear	None	NR	NR	NR	162
Galligioni (1996)	60	Unclear	None	NR	NR	NR	NR
Hoover (1993)	41	4 (1 patient), 3 (40 patients), 1 (1 patient)	None	NR	82	NR	41
Gray (1989, 1988)	129	NR	1	NR	NR	NR	NR
Adler (1987)	24	≤ 24 / Unclear	This study did not report on adverse events				
Souter (1981)	34	1	This study did not report on adverse events				
Embleton (1978), McIllmurray (1977)	8	1	None	NR	4	NR	NR

*NR* not reported.

°31 patients received vaccination; 20 of these patients were randomized to the vaccine group and 11 additional patients were placed in the vaccine group in a de-randomized fashion.

^†^Bota (2018) was the only study to provide information on AEs for control; 8 serious AEs, 58 total AEs in control group.

*in the study by Bota et al. (2018) it was unclear how many cycles of the 2 autologous cell and 3 allogeneic cell vaccines were provided.

^±^although only 150 patients were considered “analyzable” in the study by Harris (2000), 205 were vaccinated.

#### Efficacy

Overall response was reported in two studies in which vaccinated patients had measurable disease at the time of vaccination (Supplementary Fig. 1)^14,19^. There was no significant difference in overall response between the vaccine and control groups (RR 1.86; 95% CI 0.48–8.16), however data remains limited. There were no studies which reported complete response.

### Secondary outcomes: overall and disease-free survival

**Figure 2:**
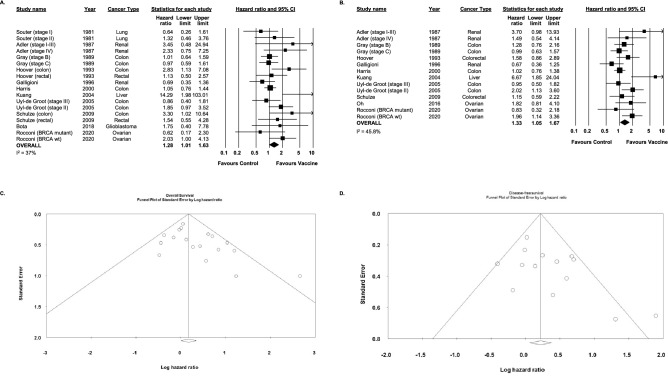
(**A**,**B**) Hazard ratio and accompanying 95% confidence interval for overall survival (**A**) and disease-free survival (**B**) in randomized control trials of patients treated with autologous whole cell vaccines for solid tumors. (**C**) Funnel plot for studies reporting on overall survival. (**D**) Funnel plot for studies reporting on disease-free survival.

Thirteen studies reported on OS (Fig. 2a). Cumulatively, vaccination was associated with a statistically significant improvement in OS (HR 1.28, 95% CI 1.01–1.63 I^2^ = 37%). Similarly, in the ten studies in which DFS was reported, survival was improved in patients receiving vaccination (HR 1.33, 95% CI 1.05–1.57 I^2^ = 45.8%, Fig. 2b). Meta-bias assessment suggested publication bias for overall survival (Fig. 2c, Egger’s regression intercept 1.36, 95% CI 0.13–2.59, p < 0.05) but not disease-free survival (Fig. 2d, Egger’s regression intercept 1.76, 95% CI 0.12–3.64, p = 0.06).

### Secondary outcomes: correlative immune assays

Eleven trials reported some form of immune assay (Table 4). The most frequently employed assay was a delayed type hypersensitivity (DTH) response, which was carried out in eight of 14 total trials. Of these eight trials, seven reported positive DTH responses in one or more patients and one study did not observe any DTH responses. In five of the seven trials patients who had a DTH response were more likely to have a better clinical outcome. The two remaining trials did not correlate responses with clinical outcomes. Other types of assays performed included an ELISPOT assay as well as various phenotypic analyses, stimulation tests, and counts of cell populations.

**Table 4:** Immune Assays.

Study	DTH/ELISPOT assays				ICS	Other immune assays?	Phenotypic analysis?
	Assay	Stimulus	Positive responses?	Correlation with clinical outcomes?			
Oh (2020) Oh (2016)	ELISPOT	Whole Cell	Yes	NR	No	No	No
Bota (2018)	Neither	NA	NA	NA	No	CD3/CD4 + count; maximal and end of treatment	Yes
Schulze (2009)	Neither	NA	NA	NA	No	No	No
Peng (2006)	DTH	Lysate	Yes	Yes	No	No	No
Uyl-de Groot (2005), Vermorken (1999)	DTH	Whole Cell	No	NA	No	No	No
Peng (2005)	DTH	Lysate	Yes	Yes	No	Flow cytometry; proportion of CD3, CD4, CD8, CD16, and CD56 positive cells	Yes
Kuang (2004)	DTH	Lysate	Yes	Yes	No	No	No
Harris (2000)	DTH	Whole Cell	Yes	Yes	No	No	No
Galligioni (1996)	DTH	Whole Cell	Yes	NR	No	No	No
Hoover (1993)	DTH	Whole Cell	Yes	NR	No	No	No
Gray (1989, 1988)	Neither	NA	NA	NA	No	No	No
Adler (1987)	DTH	Whole Cell	Yes	Yes	No	In vitro lymphocyte stimulation, immune response to tuberculin	No
Souter (1981)	Neither	NA	NA	NA	No	No	No
Embleton (1978), McIllmurray (1977)	Neither	NA	NA	NA	No	In vitro cytotoxicity and leukocyte responses to stimulants PHA, PWM, ConA	No

*DTH* delayed type hypersensitivity, *ICS* intracellular cytokine staining, *ELISPOT* enzyme linked immunospot, *NR* not reported, *NA* not applicable, *PHA* phytohaemagglutinin, *PMW* pokeweed mitogen, *ConA* concanavalin A.

### Tertiary outcomes: health utility and economic assessment

Measures of quality of life and economic value of the autologous whole cell vaccine treatment were reported in one study (7%). Uyl-de Groot (2005) reported 5.51 quality adjusted life years (QALYs) in patients receiving AWCV compared to 4.65 in patients in the control group (confidence intervals and whether study was reporting mean or median was unclear). With these estimates, the cost per QALY of the AWCV was US $22,561^22^.

### Subgroup analyses

Statistically significant differences were not observed between different disease sites with respect to improvement in overall or disease-free survival (Supplemental Fig. 2). Similarly, there was no statistically significant differences in overall or disease free survival based on the adjuvant employed (Supplemental Fig. 3). Due to insufficient data other planned subgroup analyses were not performed, including fresh vs frozen vaccine (all studies used fresh vaccine); single vs multidose (only two studies clearly reported delivering only a single dose); and centralized vs non-centralized manufacturing (only two studies reported non-centralized manufacturing).

**Figure 3:**
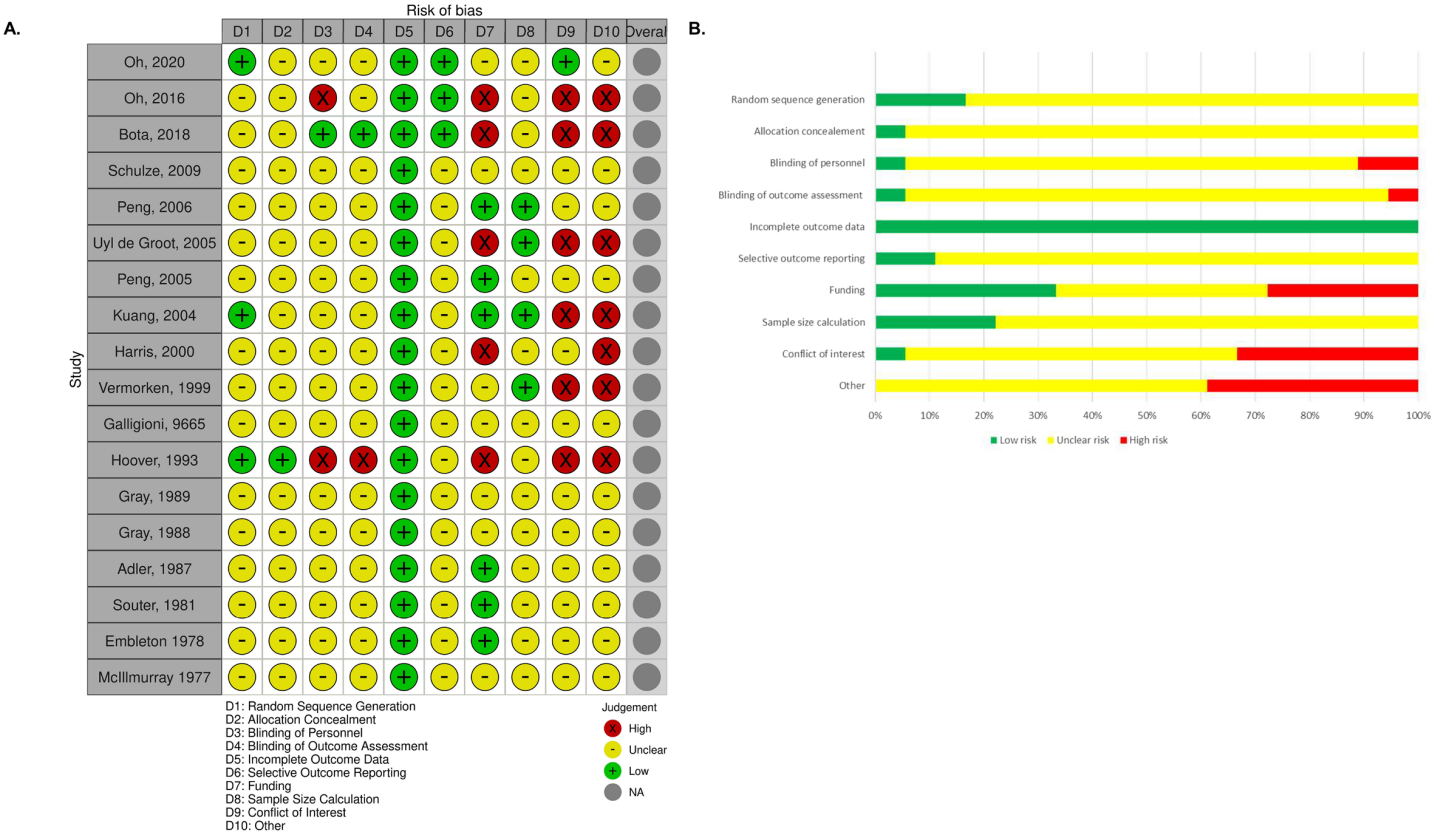
(**A**,**B**) Risk of Bias within individual studies (**A**) and across studies (**B**).

### Risk of bias

The majority of the studies included in the review were at an unclear risk of bias (Fig. 3). Fifteen out of eighteen manuscripts did not provide details on their random sequence generation, while the methods for allocation and blinding of personnel were not specific in seventeen and fifteen of the eighteen papers respectively. Pre-specified protocols were referenced in two studies, making it difficult to ascertain whether selective outcome reporting occurred. Furthermore, details of sample size calculations were provided in a minority (n = 4) of studies. The area with the greatest clear risk of bias was conflict of interest with six studies having at least one author with a significant conflict. Industry sponsorship was acknowledged in five manuscripts while seven additional manuscripts did not clearly report sources of funding.

## Discussion

Disseminated disease remains the major cause of cancer-specific mortality following the operative management of solid tumors, despite advances in adjuvant therapies^1,23–25^. Here we show that autologous whole cell vaccination represents a strategy that is safe and can extend survival in patients who have undergone tumor resection. The results of this systematic review support further investigation into these therapies.

Across 14 randomized clinical trials, 698 patients received at least one dose of autologous whole cell vaccine with only five reported high grade (grade III+) adverse events. Four of the high-grade AEs were reported in a single study of glioblastoma patients that also reported a total of 8 in the control group. This outcome suggests that concurrent therapies or the disease site (brain) of this patient cohort, rather than the vaccine itself, were contributing factors^14^. In the majority of the remaining studies, concurrent therapies were not provided (see Table 2) and AEs were only listed for the vaccination group. Thus, most of the AEs that were directly attributed to vaccination were mild, taking the form of fevers or injection site reactions, and not requiring medical intervention (see Table 3). In one study, however, a high grade AE was observed in the form of renal failure secondary to deposition of immune complexes in the glomerulus^20^. It should be noted that the remaining patients in this trial were not reported to develop similar AEs and the adjuvant, BCG, has been used extensively since without documented serious AEs^18,22,26,27^. Overall the incidence of high grade adverse events noted in our study is significantly less than what has been described for patients receiving checkpoint blockade or chemotherapy as a component of the treatment of solid tumors^28–30^. Integrated analysis of AEs between studies was complicated by inconsistent reporting metrics with some articles reporting the total number of AEs occurring and others reporting the number of patients experiencing an AE. While some of the studies captured in our review predate the publication of current standardized reporting practices, adherence to these conventions will enable better cross-study comparison in future works^31,32^.

In addition to safety, the other primary outcome in our systematic review was efficacy based on clinical response. This was included as a primary outcome because the protocol published for this systematic review was also designed for use in a study of autologous cell vaccines in patients with hematologic malignancies^6^. This metric is of limited value in the current manuscript given that the majority of studies employed autologous vaccines in the adjuvant setting in the context of patients who had previously undergone curative tumor resection. Thus, patients only had measurable disease at the time of vaccination in two studies limiting useful assessment of clinical response in the current manuscript. The cumulative risk ratio for overall response across all studies was 1.86 (95% CI 0.48 8.16, Supplemental Fig. 1). The two studies which reported on overall response were conducted in drastically different time periods in disparate malignancies^14,19^. Patients with measurable disease that is not removed by surgery typically represent advanced disease. Therefore, clinical response in the context of solid tumors is more likely to be reported in the context of advanced disease which may be inherently more resistant to immunotherapy^33–35^.

Although analyzed as a secondary outcome, the results obtained for survival outcomes may be more useful in terms of describing the efficacy of these therapeutics as there is more data available for this outcome. Overall, autologous whole cell vaccines were shown to increase the overall and disease-free survival relative to the respective randomized control populations (Fig. 2). The beneficial effects of vaccination on survival were significantly greater in patients with earlier stage disease in three studies where this comparison was made^19,21,22^. Larger tumors have also been found to be more immunosuppressive and it is possible that more locally advanced disease has further dampened the immune response^33–35^. Although the opposite trend was observed by Souter et al., the overall survival was not significantly different between treatment and control groups for either stage I or II lung cancer^36^. An interesting observation made by both Hoover et al. and Schulze et al. is the superior performance of autologous whole cell vaccines in cancers of the colon relative to those of the rectum^17,18^. The immunobiology of this phenomenon remains unclear. There is an emerging consensus that right sided colonic tumors typically exhibit higher antigenic loads and better responses to immunotherapies than left and the highly immunogenic DNA mismatch repair deficient (MMRD) tumors are more prevalent on the right, providing one possible explanation^37,38^. There were no statistically significant differences in overall survival based on the adjuvants employed, although experience with adjuvants such as NDV were quite limited and there was significant variability in these subgroups (Supplemental Fig. 3). No study captured in this review reported on differences in outcomes associated with number of doses given, although evidence for benefit from more doses has been observed in the use of autologous whole cell vaccines for hematologic malignancies^39–42^.

Recently, the results of a phase IIb RCT investigating the use of an autologous cell vaccine as an adjuvant to standard of care surgery and chemotherapy in stage III/IV high grade serous, endometroid, and clear cell ovarian cancers were published. This trial demonstrated a modest improvement in recurrence free survival in the group receiving the vaccine although this did not reach statistical significance (p = 0.078). Nevertheless, no grade 3 or 4 adverse events were attributed to the vaccine which is in agreement with out findings^43^.

In most instances where an immune assay was carried out positive results were obtained, at least in some patients (see Table 4). The most frequently employed investigation was the DTH assay. This assay is relatively simple and amenable to use in clinical trials with large volumes of patients. DTH responses are commonly used in other approaches to cancer vaccination and thus allow for a comparison to other therapies as well^44,45^. Although the DTH results appeared to correlate well with clinical outcomes in this study, the assay is limited in that it does not provide information on the immune subpopulations mediating the response and is less useful in quantifying the magnitude of response beyond a binary answer.

A significant limitation to this study is the variability in terms of protocols and time periods during which trials were conducted. Half of the 18 reports captured in this systematic review were published prior to the year 2000 with the remaining being published subsequently. Technical knowledge, reporting standards, and our ability to accurately assess residual disease have advanced considerably. Knowledge of tumor immunology and cancer immunotherapy has also expanded significantly over the timeframe in which studies are published. It is therefore less informative to rely on quantitative outcomes reported in earlier studies than it is to consider the overall trends that were established. The variability in disease sites investigated, while reducing the power of our report to make conclusions regarding any one tumor type, provides a broader overview of the efficacy of autologous whole cell vaccination. While comparison between outcomes in different disease sites can be performed based on our analysis (Supplemental Fig. 2), it is most reliable in studies which carried out comparisons in the setting of a single trial.

Of the 808 enrolled patients, 698 received at least one dose of vaccine representing a successful vaccination rate of 86%. This rate is significantly better than what was observed in our systematic review of autologous whole cell vaccination in hematologic malignancies (58%) but still suggests that the ability to manufacture vaccines represents a barrier in the context of solid tumors^12^. In the current study, the main reason for patients not receiving vaccination was withdrawal or being lost to follow-up followed by post-operative complications / death and patients becoming ineligible^18,20,36,46–48^. Unlike our systematic review of autologous whole cell vaccination in hematologic malignancies manufacturing challenges represented a reason for which only a minority of patients were not vaccinated^12^.

Overall, our systematic review and meta analysis indicate that autologous whole cell vaccination represents a safe strategy that can potentially extend overall survival when used as an adjuvant to surgical management of solid tumors. Our review was limited by the broad publication range, limited power to make conclusions in any one disease site, and inconsistencies in reporting standards across studies. Given the significant advances that have been made in basic tumor immunobiology and immunotherapy as a field in the last decade, there is strong rationale to continue exploring autologous whole cell vaccines as an adjuvant strategy to the surgical management of solid tumors.

## Supplementary Information

Supplementary Information 1.

Supplementary Information 2.

Supplementary Information 3.

Supplementary Information 4.

Supplementary Information 5.

## Data Availability

All data generated or analysed during this study are included in this published article [and its supplementary information files].
