# Supplementary material for: Safety and efficacy of autologous cell vaccines in solid tumors: a systematic review and meta-analysis of randomized control trials
Source: Sci Rep. 2023 Feb 27;13:3347. doi: 10.1038/s41598-023-29630-9 (PMC9971202; doi:10.1038/s41598-023-29630-9)
Supplement: Supplementary file 2 — Supplementary Information 2. [file 41598_2023_29630_MOESM2_ESM.docx]

**Appendix 2: Search Strategy**

Database: Ovid MEDLINE(R) ALL <1946 to August 06, 2020>

Search Strategy:

--------------------------------------------------------------------------------

1 Cancer Vaccines/ (13442)

2 (cancer adj5 vaccin*).tw. (10489)

3 ((tumor or tumour or oncoly*) adj2 vaccin*).tw. (4110)

4 (cancer vaccin* or oncoly* vaccin* or tumor vaccin* or tumour vaccin* or tumor cell vaccin* or tumour cell vaccin*).kw. (990)

5 (adagloxad simolenin or adegramotide or algenpantucel L or baltaleucel T or belagenpumatucel L or biropepimut Sor crs 207 or dasiprotimut T or dorgenmeltucel L or dpx survivac or eltrapuldencel T or galinpepimut S or lapuleucel T).tw,kw. (48)

6 (lymphoma vaccine or melanoma vaccine or mitumprotimut T or modified vaccinia virus Ankara 5T4 vaccine or nelatimotide or nelipepimut S or onamelatucel L or ranagengliotucel T or rindopepimut).tw,kw. (275)

7 (rocapuldencel T or seviprotimut L or sipuleucel T or tergenpumatucel L or tisagenlecleucel T or vadacabtagene leraleucel or vesigenurtucel L or viagenpumatucel L).tw,kw. (576)

8 or/1-7 (21764)

9 (allogeneic not autologous).tw,kw. (50779)

10 8 not 9 (21246)

11 (whole adj2 cell* adj3 vaccin*).tw. or whole cell* vaccin*.kw. (1978)

12 (autologous and vaccin* and cell*).tw,kf. (3429)

13 ((autologous adj3 infusion*) and cell*).tw. (627)

14 (personal* adj2 vaccin*).tw. (436)

15 (autologous adj3 vaccin*).tw. (904)

16 autologous vaccin*.kw. (7)

17 or/11-16 (6421)

18 exp neoplasms/ (3348126)

19 (cancer or tumor* or tumour* or neoplasm* or malignan* or glioblastoma or carcinoma or melanoma or leuk?em* or myeloma*).tw,kw. (3467277)

20 18 or 19 (4362438)

21 17 and 20 (3431)

22 10 or 21 (22485)

23 randomized controlled trial.pt. (510873)

24 controlled clinical trial.pt. (93789)

25 random*.tw. (1148826)

26 placebo.ab. (209925)

27 trial.ti. (222806)

28 (phase 1 or phase 2 or phase II or phase 3 or phase III).tw,kw. (117801)

29 clinical trial*.pt. (575475)

30 (clinical adj2 (trial* or results*)).tw. not review.pt. (302224)

31 or/23-30 (1888348)

32 22 and 31 (4131)

33 exp animals/ not humans/ (4723823)

34 32 not 33 (3888)

Database: Embase Classic+Embase <1947 to 2020 August 06>

Search Strategy:

--------------------------------------------------------------------------------

1 exp *cancer vaccine/ or cancer vaccine/ (16826)

2 (cancer adj5 vaccin*).tw. (14550)

3 (adagloxad simolenin or adegramotide or algenpantucel L or baltaleucel T or belagenpumatucel L or biropepimut Sor crs 207 or dasiprotimut T or dorgenmeltucel L or dpx survivac or eltrapuldencel T or galinpepimut S or lapuleucel T).tw. (122)

4 (lymphoma vaccine or melanoma vaccine or mitumprotimut T or modified vaccinia virus Ankara 5T4 vaccine or nelatimotide or nelipepimut S or onamelatucel L or ranagengliotucel T or rindopepimut or rocapuldencel T or seviprotimut L or sipuleucel T).tw. (1445)

5 (tergenpumatucel L or tisagenlecleucel T or vadacabtagene leraleucel or vesigenurtucel L or viagenpumatucel L).tw. (25)

6 ((tumor or tumour) adj2 vaccin*).tw. (5200)

7 oncoly* vaccin*.tw. (405)

8 or/1-7 (29035)

9 (allogeneic not autologous).tw. (80431)

10 8 not 9 (28356)

11 (whole adj2 cell* adj3 vaccin*).tw. (2286)

12 (autologous and cell* and vaccin*).tw. (5046)

13 (autologous adj3 vaccin*).tw. (1342)

14 (personal* adj2 vaccin*).tw. (815)

15 ((autologous adj3 infusion*) and cell*).tw. (1075)

16 or/11-15 (9102)

17 exp *neoplasm/ (3636244)

18 (cancer or tumor* or tumour* or neoplasm* or malignan* or glioblastoma or carcinoma or melanoma or leuk?em* or myeloma*).tw. (4859927)

19 17 or 18 (5548152)

20 16 and 19 (5313)

21 10 or 20 (30890)

22 random*.tw. or placebo*.mp. or double-blind*.tw. (1842786)

23 trial.ti. (314059)

24 exp *clinical trial/ (53369)

25 controlled clinical trial/ (465291)

26 randomized controlled trial/ (618242)

27 (phase 1 or phase 2 or phase II or phase 3 or phase III).tw. (223407)

28 or/22-27 (2278406)

29 21 and 28 (5035)

30 (exp animals/ or exp nonhumans/) not exp humans/ (5460421)

31 abstract.pt. (3841907)

32 29 not (30 or 31) (3422)

Database: EBM Reviews - Cochrane Central Register of Controlled Trials <June 2020>

Search Strategy:

--------------------------------------------------------------------------------

1 Cancer Vaccines/ (311)

2 (cancer adj5 vaccin*).tw. (1159)

3 ((tumor or tumour or oncoly*) adj2 vaccin*).tw. (301)

4 (cancer vaccin* or oncoly* vaccin* or tumor vaccin* or tumour vaccin* or tumor cell vaccin* or tumour cell vaccin*).kw. (144)

5 (adagloxad simolenin or adegramotide or algenpantucel L or baltaleucel T or belagenpumatucel L or biropepimut Sor crs 207 or dasiprotimut T or dorgenmeltucel L or dpx survivac or eltrapuldencel T or galinpepimut S or lapuleucel T).tw,kw. (37)

6 (lymphoma vaccine or melanoma vaccine or mitumprotimut T or modified vaccinia virus Ankara 5T4 vaccine or nelatimotide or nelipepimut S or onamelatucel L or ranagengliotucel T or rindopepimut).tw,kw. (105)

7 (rocapuldencel T or seviprotimut L or sipuleucel T or tergenpumatucel L or tisagenlecleucel T or vadacabtagene leraleucel or vesigenurtucel L or viagenpumatucel L).tw,kw. (155)

8 or/1-7 (1786)

9 (allogeneic not autologous).tw,kw. (4265)

10 8 not 9 (1710)

11 (whole adj2 cell* adj3 vaccin*).tw. or whole cell* vaccin*.kw. (375)

12 (autologous and vaccin* and cell*).tw. (481)

13 ((autologous adj3 infusion*) and cell*).tw. (241)

14 (personal* adj2 vaccin*).tw. (152)

15 (autologous adj3 vaccin*).tw. (211)

16 autologous vaccin*.kw. (0)

17 or/11-16 (1252)

18 exp neoplasms/ (78081)

19 (cancer or tumor* or tumour* or neoplasm* or malignan* or glioblastoma or carcinoma or melanoma or leuk?em* or myeloma*).tw,kw. (224220)

20 18 or 19 (237248)

21 17 and 20 (629)

22 10 or 21 (2031)

23 abstract.pt. (12)

24 conference abstract.pt. (16797)

25 journal conference abstract.pt. (157141)

26 abstract.so. (1020)

27 or/23-26 (174716)

28 22 not 27 (1603)
