# Supplementary material for: Safety and efficacy of autologous cell vaccines in solid tumors: a systematic review and meta-analysis of randomized control trials
Source: Sci Rep. 2023 Feb 27;13:3347. doi: 10.1038/s41598-023-29630-9 (PMC9971202; doi:10.1038/s41598-023-29630-9)
Supplement: Supplementary file 3 — Supplementary Information 3. [file 41598_2023_29630_MOESM3_ESM.pdf]

Study name	Year	Cancer Type	Statistics for each study		
			Risk ratio	Lower limit	Upper limit
Adler	1987	Renal	0.67	0.05	9.19
Bota	2018	Glioblastoma	3.00	0.50	17.95
**OVERALL**			**1.86**	**0.42**	**8.16**

Risk ratio and 95% CI

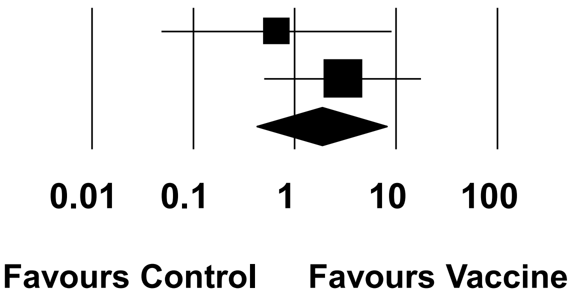

Supplemental Figure 1. Overall Response and accompanying 95% confidence interval for patients treated with autologous whole cell vaccines for solid tumors.
