# Supplementary material for: Safety and efficacy of autologous cell vaccines in solid tumors: a systematic review and meta-analysis of randomized control trials
Source: Sci Rep. 2023 Feb 27;13:3347. doi: 10.1038/s41598-023-29630-9 (PMC9971202; doi:10.1038/s41598-023-29630-9)
Supplement: Supplementary file 4 — Supplementary Information 4. [file 41598_2023_29630_MOESM4_ESM.pdf]

A.

Study name	Year	Cancer Type	Statistics for each study: Hazard ratio	Lower limit	Upper limit
Gray (stage B)	1989	Colon	1.01	0.64	1.59
Gray (stage C)	1989	Colon	0.97	0.59	1.61
Hoover (colon)	1993	Colon	2.83	1.13	7.08
Harris	2000	Colon	1.05	0.76	1.44
Uyl-de Groot (stage II)	2005	Colon	1.85	0.97	3.52
Uyl-de Groot (stage III)	2005	Colon	0.86	0.40	1.81
Schulze (colon)	2009	Colon	3.30	1.02	10.64
**Colon OVERALL**			**1.25**	**0.93**	**1.68**
Bota	2018	Glioblastoma	1.75	0.40	7.78
**Glioblastoma OVERALL**			**1.75**	**0.40**	**7.78**
Kuang	2004	Liver	14.29	1.98	103.01
**Liver OVERALL**			**14.29**	**1.98**	**103.01**
Souter (stage I)	1981	Lung	0.64	0.26	1.61
Souter (stage II)	1981	Lung	1.32	0.46	3.76
**Lung OVERALL**			**0.88**	**0.44**	**1.76**
Rocconi (BRCA mutant)	2020	Ovarian	0.62	0.17	2.30
Rocconi (BRCA wt)	2020	Ovarian	2.03	1.00	4.13
**Ovarian OVERALL**			**1.28**	**0.41**	**3.97**
Hoover (rectal)	1993	Rectal	1.13	0.50	2.57
Schulze (rectal)	2009	Rectal	1.54	0.55	4.28
**Rectal OVERALL**			**1.27**	**0.67**	**2.42**
Adler (stage I-III)	1987	Renal	3.45	0.48	24.94
Adler (stage IV)	1987	Renal	2.33	0.75	7.25
Galligioni	1996	Renal	0.69	0.35	1.36
**Renal OVERALL**			**1.41**	**0.50**	**3.96**

Hazard ratio and 95% CI

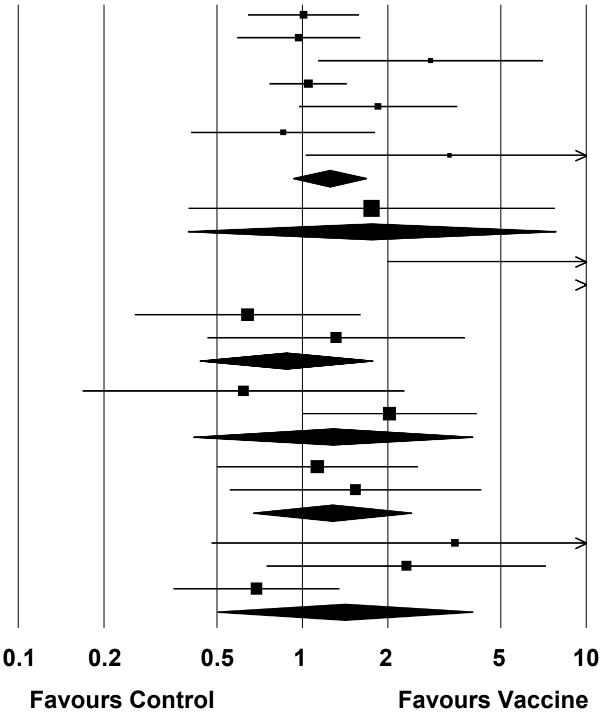

B.

Study name	Year	Cancer Type	Statistics for each study: Hazard ratio	Lower limit	Upper limit
Gray (stage B)	1989	Colon	1.28	0.76	2.16
Gray (stage C)	1989	Colon	0.99	0.63	1.57
Harris	2000	Colon	1.02	0.76	1.38
Uyl-de Groot (stage II)	2005	Colon	2.02	1.13	3.60
Uyl-de Groot (stage III)	2005	Colon	0.95	0.50	1.82
**Colon OVERALL**			**1.15**	**0.91**	**1.46**
Hoover	1993	Colorectal	1.58	0.86	2.89
Schulze	2009	Colorectal	1.15	0.59	2.22
**Colorectal OVERALL**			**1.37**	**0.88**	**2.13**
Kuang	2004	Liver	6.67	1.85	24.04
**Liver OVERALL**			**6.67**	**1.85**	**24.04**
Oh (original)	2016	Ovarian	1.82	0.81	4.10
Rocconi (BRCA mutant)	2020	Ovarian	0.83	0.32	2.18
Rocconi (BRCA wt)	2020	Ovarian	1.96	1.14	3.36
**Ovarian OVERALL**			**1.61**	**1.02**	**2.55**
Adler (stage I-III)	1987	Renal	3.70	0.98	13.93
Adler (stage IV)	1987	Renal	1.49	0.54	4.14
Galligioni	1996	Renal	0.67	0.36	1.25
**Rena OVERALL**			**1.35**	**0.52**	**3.52**

Hazard ratio and 95% CI

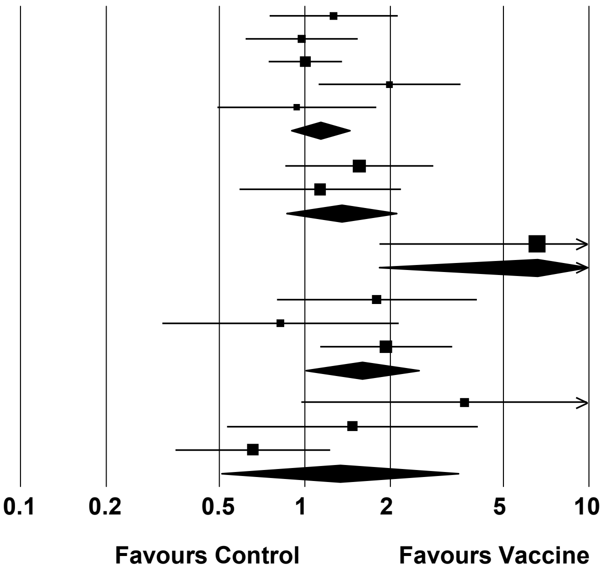

Supplemental Figure 2. (**A** and **B**) Overall Survival (A) and Disease-Free Survival (B) based on disease site.
