# Supplementary figures and images for: Safety and efficacy of autologous cell vaccines in solid tumors: a systematic review and meta-analysis of randomized control trials
Source: Sci Rep. 2023 Feb 27;13:3347. doi: 10.1038/s41598-023-29630-9 (PMC9971202; doi:10.1038/s41598-023-29630-9)

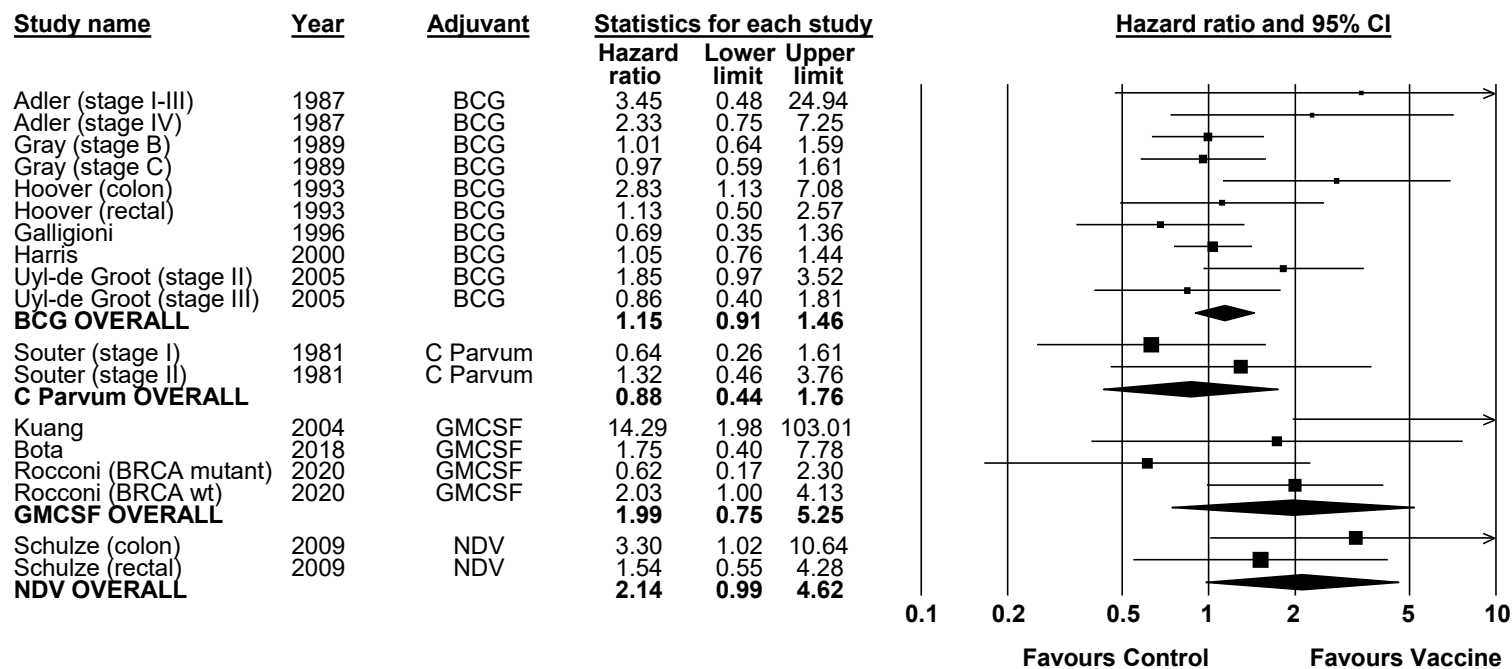

**Supplemental Figure 3.** Overall Survival based on adjuvant.

Supplement: Supplementary file 5 — Supplementary Information 5. [file 41598_2023_29630_MOESM5_ESM.pdf]
